# Investigating the Production of Antimicrobial Nanoparticles by *Chlorella vulgaris* and the Link to Its Loss of Viability

**DOI:** 10.3390/microorganisms10010145

**Published:** 2022-01-11

**Authors:** Munirah F. Aldayel, Mayyadah A. Al Kuwayti, Nermin A. H. El Semary

**Affiliations:** 1 Department of Biological Sciences, College of Science, King Faisal University, Al Hofuf 31982, Saudi Arabia; maldayel@kfu.edu.sa (M.F.A.); Malkuwaiti@kfu.edu.sa (M.A.A.K.); 2 Botany and Microbiology Department, Faculty of Science, Helwan University, Cairo 11795, Egypt

**Keywords:** antibacterial bioassay, *Chlorella vulgaris*, nanogold, nanosilver, RNA extraction

## Abstract

*Chlorella vulgaris* from Al-Ahsa, KSA was proved to be an active silver and gold nanoparticle producer. Nanogold and nanosilver particles were characterized using UV-visible spectroscopy, Fourier-transform infrared spectroscopy, and scanning electronmicroscopy. Both nanoparticles were used in the antimicrobial bioassay. The two nanoparticles showed antibacterial activities, with the silver nanoparticles being the most effective. To investigate the argumentative nature of their biosynthesis (i.e., whether it is a biotic or abiotic process), we isolated total ribonucleic acid RNA as an indicator of vitality. RNA was completely absent in samples taken after one week of incubation with silver nitrate and even after one or two days. However, successful extraction was only achievable in samples taken after incubation for one and four hours with silver nitrate. Most importantly, the gel image showed recognizable shearing of the nucleic acid after 4 h as compared to the control. An assumption can be drawn that the synthesis of nanoparticles may start biotically by the action of enzyme(s) and abiotically by action of reducing entities. Nonetheless, with prolonged incubation, excessive nanoparticle accumulation can be deadly. Hence, their synthesis continues abiotically. From the RNA banding profile, we suggest that nanosilver production starts both biotically and abiotically in the first few hours of incubation and then continues abiotically. Nanosilver particles proved to have more of an antimicrobial impact than nanogold and hence are recommended for different applications as antibacterial agents.

## 1. Introduction

Microalgae are considered as significant bio-factories for the treatment of wastewater, the production of biofuel, the manufacturing of a variety of commercially valuable products, and the synthesis of nanoparticles. The synthesis of nanoparticles from biological sources has appeared recently as a potential substitute to the typical physical and chemical methods. Due to their cost-effectiveness and environmental friendliness, some microorganisms, such as bacteria, algae, and fungi, have been successfully implemented as prospective prolific source of eco-friendly biogenic nanoparticles. For example, different algal species belonging to different algal groups such as *Chlorophyta*, *Haptophyta*, and *Ochrophyta* were reported to biosynthesize nanoparticles [1,2]. According to a recent review by Jacob et al. [3], their ease of growth and ability to live in extreme conditions make them plausible candidates for large-scale synthesis of nanoparticles. The ability to biosynthesize nanoparticles is suggested to be a mechanism by which the microalgae detoxify excessive bulky metals in their niche which can otherwise be toxic and hazardous [4]. The biomass itself can be used as well as the cell free supernatants. 

Microalgae can be regarded as a potent nano-factory, capable of manufacturing diverse nanoparticles such as silver, gold, cadmium, and platinum [5]. Several methods for generating metallic nanoparticles from microalgae and their related aqueous salt solutions have been proposed. For the biosynthesis of silver and gold nanoparticles, Li et al. [6] utilized a fine powder of *Spirogyra insignis* (Charophyta). It has been shown that cell-free supernatants of cyanobacteria and chlorophyta species can be used to synthesize silver nanoparticles [7]. Interestingly, Agarwal et al. pointed out in their review that gold shape-directing high molecular weight protein and silver nanoparticle low molecular weight proteins contained in the biomass of microalgae are responsible for reducing gold and silver ions into their neutral metallic nanoform [5]. In addition, silver nanoparticle manufacturing has been demonstrated in different algal species such as *Chlorella vulgaris*, *Spirulina platensis*, and *Lyngbya majuscula* [8].

The bio-based synthesis has been proposed as a means of producing nanoparticles. The basic concept of this kind of synthesis is to transform metal ions into nanoparticles or bioactive products with high reducing power via a cell factory [9]. Algae include a wide array of bioactive compounds (for example, polysaccharides, vitamins, proteins, lipids, and pigments) which can be used to synthesize various metals nanoparticles by activating the detoxifying machinery in both living cells and cellular extracts [6]. Several studies have addressed the synthesis of nanomaterials [10,11]. Metallic nanoparticles can be generated using one of two bio-based synthesis methods; the first one is an *in vivo* biosynthesis by which the metal ions are delivered into cells or onto the cell surface, where they are then transformed into metallic nanomaterials by a particular enzyme via the metallic detoxification machinery. The second biosynthesis method is an *in vitro* by which metallic ions are collected and converted into metallic nanomaterials utilizing bioactive component derived from microorganisms [12]. Nonetheless, there is no consensus in the literature about the exact route the biosynthesis can follow. Therefore, and in order to fill in the gap in our knowledge, we attempt to investigate the of the biotic and abiotic nanosilver synthesis.

With regard to antimicrobial activity of nanoparticles, Hafez (2013) showed that nano-silver particles produced by some microalgae had antimicrobial activity [13]. He used aqueous extract from the algae *Chroococcus disperses* and *Chlorella vulgaris* for production of nanosilver and tested them against two plant pathogens (*Pseudomonas flavescens* and *Erwinia amylovora)* in addition to several plant pathogenic fungi. The results showed that high antibacterial activity was obtained by the produced nanosilver and the inhibition zones exceeded that of control antibiotic disc. Similar results were obtained for the antifungal test. The fact that microalgae, such as *Chlorella vulgaris*, grow rapidly in relatively inexpensive growth media makes them a more plausible cost-effective candidates for nanoparticles biosynthesis. Here in the present study, we attempted to evaluate and compare the antimicrobial activity of both nanogold and nanosilver biosynthesized by *Chlorella vulgaris* and to further investigate the most active nanoparticles against more pathogens.

## 2. Materials and Methods

### 2.1. The Cyanobacterial Cells

The coccoid algal monospecific culture was collected and purified using standard microalgal culturing techniques. Mid-logarithmic cultures were used for the biosynthesis of silver and gold nanoparticles.

### 2.2. Silver Nanoparticles Biosynthesis

Silver nitrate (Sigma-Aldrich, St. Louis, MO, USA) was prepared at 10 mM concentration and kept in dark bottle. Cells of *Chlorella vulgaris were* washed with distilled water and centrifuged. The cells were incubated with silver nitrate solution for two weeks. The color of the outer solution turned gradually into yellow, pale brown and then dark brown. The formed silver nanoparticles were subjected to the following analyses (UV-visible spectroscopy and Fourier-Transform infrared spectrometer, FTIR).

### 2.3. Nanogold Particles Biosynthesis

*Chlorella vulgaris* mid-logarithmic culture was treated with 1500 mg/mL HAuCl_4_ solution containing Au^3+^. Immediately after the incubation, gradual slow color change was observed to occur until the complete change into a pink color. The nanoparticle suspensions produced were scanned using UV-visible spectroscopy (540 nm) and FTIR.

### 2.4. UV–Visible Spectroscopic (UV–vis) Analysis

The UV–vis spectrophotometer (Genesys10S UV–visible double beam spectrophotometer) was used. Scanned spectrum was recorded for the wavelength range of 200 nm to 600 nm.

### 2.5. FTIR Analysis

Characterization of silver nanoparticles was performed on dried biosamples of *chlorella* containing nanoparticles using Fourier-Transform infrared spectrometer (FTIR, Agilent Cory 630, Agilent Technologies, Santa Clara, CA, USA).

### 2.6. Scanning Electron Microscopy

Both aqueous external solutions containing nanosilver particles and nanogold particles were used in scanning electron microscopy. One drop of each solution was mounted on the metallic stub, left to air dry and fixed using double face adhesive carbon. The stubs were coated with gold three times using sputter, and the samples were then visualized using Joel JSM-5510LV scanning electron microscope.

### 2.7. Antibacterial Bioassay Using Disc Diffusion Method

The antibacterial potency of nanosilver and nanogold particles synthesized by *Chlorella vulgaris*, was evaluated against pathogenic bacterial strains namely; methicillin-resistant *staphylococcus aureus* (MRSA), *Staphylococcus aureus* and *Streptococcus* sp. These Gram-positive pathogenic bacteria were freshly sub-cultured prior to their assay. The sensitivity of pathogenic strains to the extract was assayed through modified Kirby Bauer disk diffusion susceptibility method [14]. Sterilized paper discs (6 mm in diameter) were saturated with 30 µL of equal concentrations of nanosilver and nanogold particles for antibacterial tests. Discs were dried and placed on the surface of inoculated nutrient agar medium with bacterial suspensions prepared in physiological saline (0.85 g NaCl in 100 mL distilled water). Plates were kept for 2 h at 4 °C to ensure diffusion of bioactive material, after that the plates were incubated at 37 °C. Control discs containing 30 µL of sterilized distilled water were left to dry, then used as negative control whereas positive control sets included discs of antibiotic Chloramphenicol at concentration of 5%. Plates were incubated for 24 h and diameter of inhibition zones (mm) were measured in triplicates and the average standard deviation was recorded. The results were compared, and the antimicrobial bioassay was further performed in a single experiment on *Escherichia coli* (gram negative bacterium) using both silver and gold nanoparticles to explore their broad-spectrum antibacterial bioactivity.

### 2.8. The Antibacterial Biovassay Using MIC/MBC

The minimum inhibitory concentration (MIC) of the synthesized AgNPs against four different bacterial pathogens (*E. coli*, *S. aureus*, MRSA, and *Streptococcus* sp.) was determined using a standard protocol [15]. Briefly, the compounds were dissolved in dimethyl sulfoxide (5% DMSO) and sterilized using a 0.2 μm Millipore membrane filter (Merck Millipore, Burlington, MA, USA). The solution was then serially diluted (two-fold in a 96-well microtiter plate) in the Mueller Hinton broth (MHB). An aliquot (5 μL) of the freshly prepared bacterial suspensions of the test organism (at 1.5 × 10^6^ colony forming units (CFU) mL^−1^) was transferred into 200 mL of sterilized MHB. After mixing, the 96-well microtiter plates were covered with a sterile plate sealer and incubated at 37 °C for 18 h under shaking. After incubation, 5 mL of the microbial cells in each well were spotted on Mueller Hinton agar plates and incubated at 37 °C for 18 h. The visible growth was monitored to determine the MIC of the particles. The experiment was repeated three times to confirm the antibacterial activity of the silver nanoparticles.

### 2.9. Chlorella vulgaris RNA Extraction

20 mL of *Chlorella vulgaris* cultures were transferred to centrifuge tubes and centrifuged at 14,000 rpm for 10 min. Pelleted cells were disrupted in liquid nitrogen in a ceramic mortar and homogenized with 500 µL of Trizol^®^ reagent (Life Technologies Corp., Carlsbad, CA, USA). Total RNA was extracted according to the manufacturer’s instructions for the reagent. Subsequently, the RNA concentrations were determined using NanoDrop™ 2000c spectrophotometer (Thermo Fisher Scientific, Waltham, MA, USA). The integrity of the extracted RNA was analyzed by electrophoresis in 1% agarose gel.

## 3. Results

### 3.1. UV-Visible Spectroscopy

**Figure 1:**
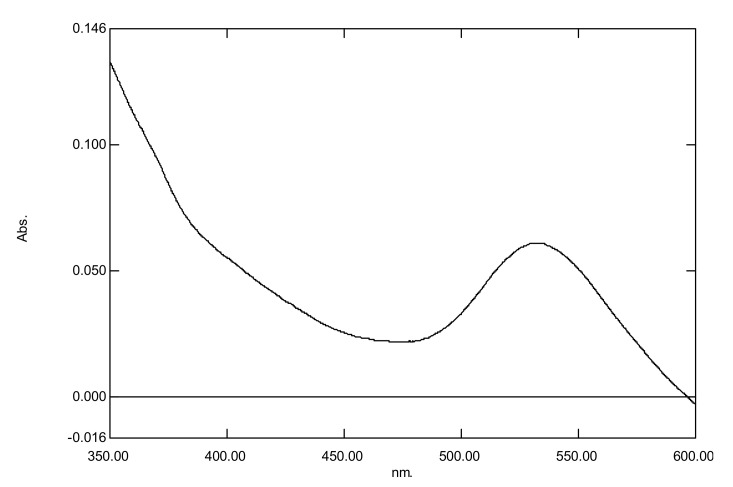
Ultra violet-visible spectrum of nanogold.

**Figure 2:**
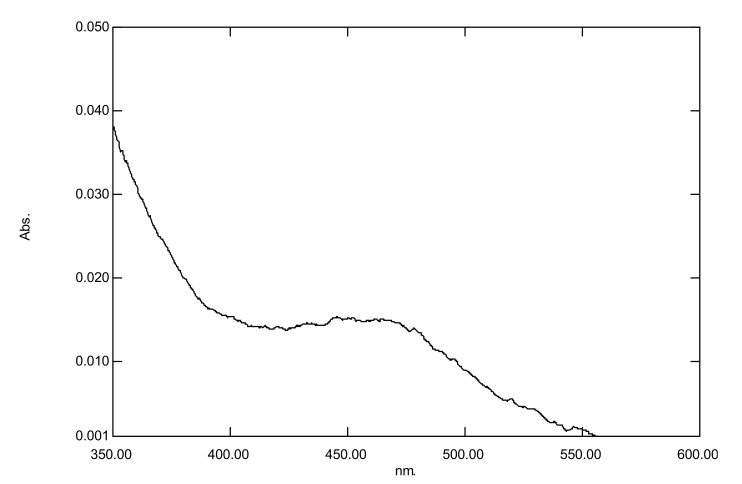
Ultra violet-visible spectrum of nanosilver.

With regard to the UV-visible spectrum of nanogold, the surface plasmon band was detected within the range of 520–550 nm (Figure 1) similar to that reported by [16]. With regard to silver nanoparticles, the surface plasmon band was found within the range of 410–450 nm (Figure 2) similar to that reported by [17]. The results showed that the nanogold plasmon resonance band was detected in the range of 510–550 nm (which is in accordance with [17].

### 3.2. FTIR

**Figure 3:**
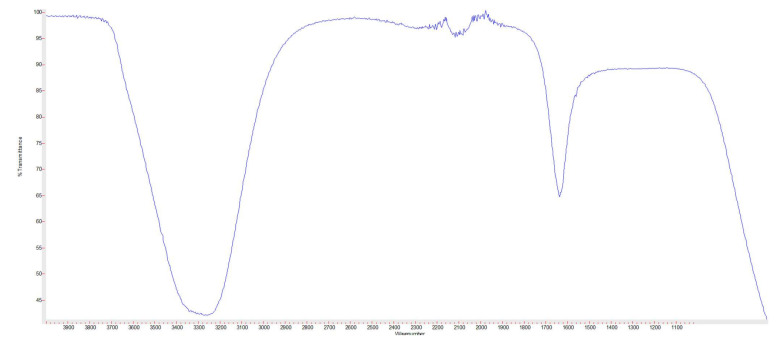
Fourier-Transform infrared (FTIR) spectrum of nanogold.

In addition, FTIR confirmed their biosynthesis by showing the characteristic bands of gold nanoparticles (Figure 3) which were similar to that reported in [17]. With regard to the FTIR, the characteristic bands of nanogold were detected at 3414.96, 2158.28, and 1640.60  cm^−1^.

**Figure 4:**
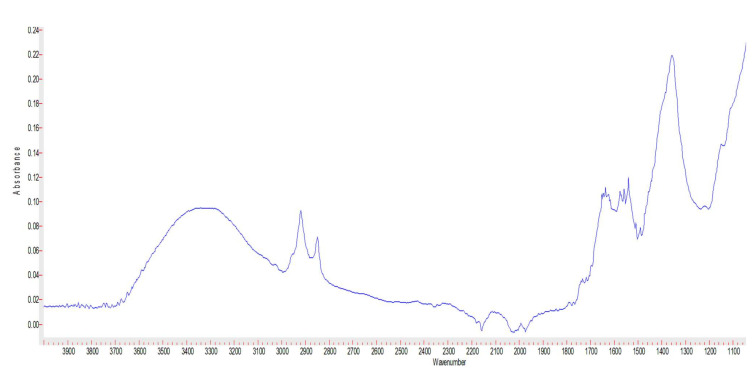
FTIR spectrum of nanosilver.

The FTIR spectra of the AgNPs. and the FTIR (Figure 4) showed the characteristic silver nanoparticles peaks at 2927, 1631, 1383 cm^−1^ and 3297 cm^−1^ [18].

### 3.3. Scanning Electron Microscopy

**Figure 5:**
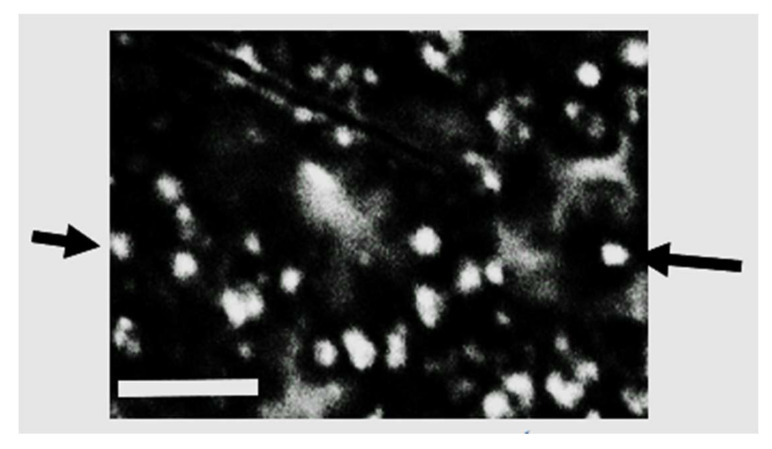
Scanning electromicrograph showing the spherical shape of biogenic nanogold particles (denoted by black arrows) (scale bar 100 nm).

**Figure 6:**
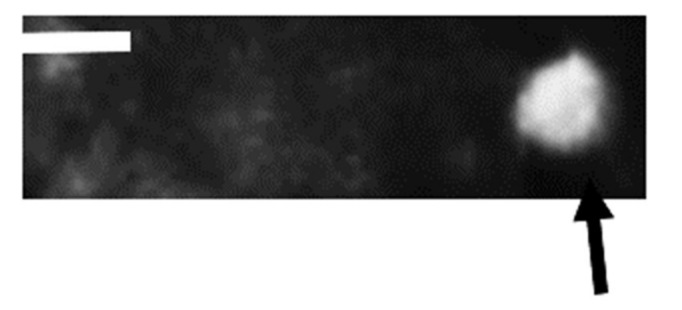
Scanning electromicrograph showing the spherical shape of biogenic nanosilver particles (denoted by black arrow) (Scale bar 100 nm).

SEM revealed the presence of spherical nanogold particles (Figure 5) with size range of 20–25 nm of uniform spherical appearance. In case of silver nanoparticles, a noticeable aggregation was frequently noticed but the individual silver nanoparticles were also abundant in the size range of 50–80 nm (Figure 6) with spherical appearance although when aggregated the aggregated particles become angular.

### 3.4. Total RNA Extraction

**Figure 7:**
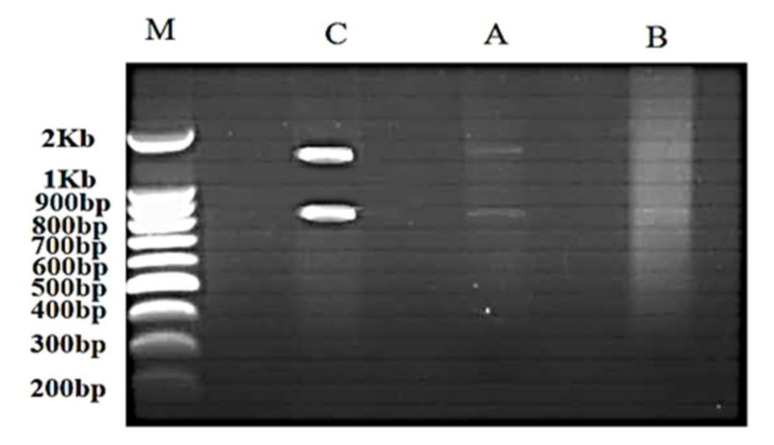
Gel electrophoresis of RNA extracted from *Chlorella vulgaris*. Extracted RNA from: untreated sample with silver nitrate (control C), *C. vulgaris* incubated with silver nitrate for one hour (A), *C. vulgaris* incubated with silver nitrate for four hours with smear formation (B); (M), molecular size marker.

Total RNA was extracted from *C. vulgaris* in order to detect cells vitality. Electrophoresis gel has been used to analyze the integrity of extracted RNA. Intact extracted RNA was detected from the control sample that was untreated with silver nitrate. However, there was a fading band for sample taken after one hour and rather “ghost” bands after four hours. Indeed, nucleic acid smearing was faintly observed for one-hour sample and intensified for the four-hour incubation (Figure 7). Furthermore, none of the extracted RNA was detected from *C. vulgaris* that was incubated with silver nitrate for six and eight hours as well as those for one day, two days, and one week.

### 3.5. Antimicrobial Bioassay Using Disc Diffusion

**Figure 8:**
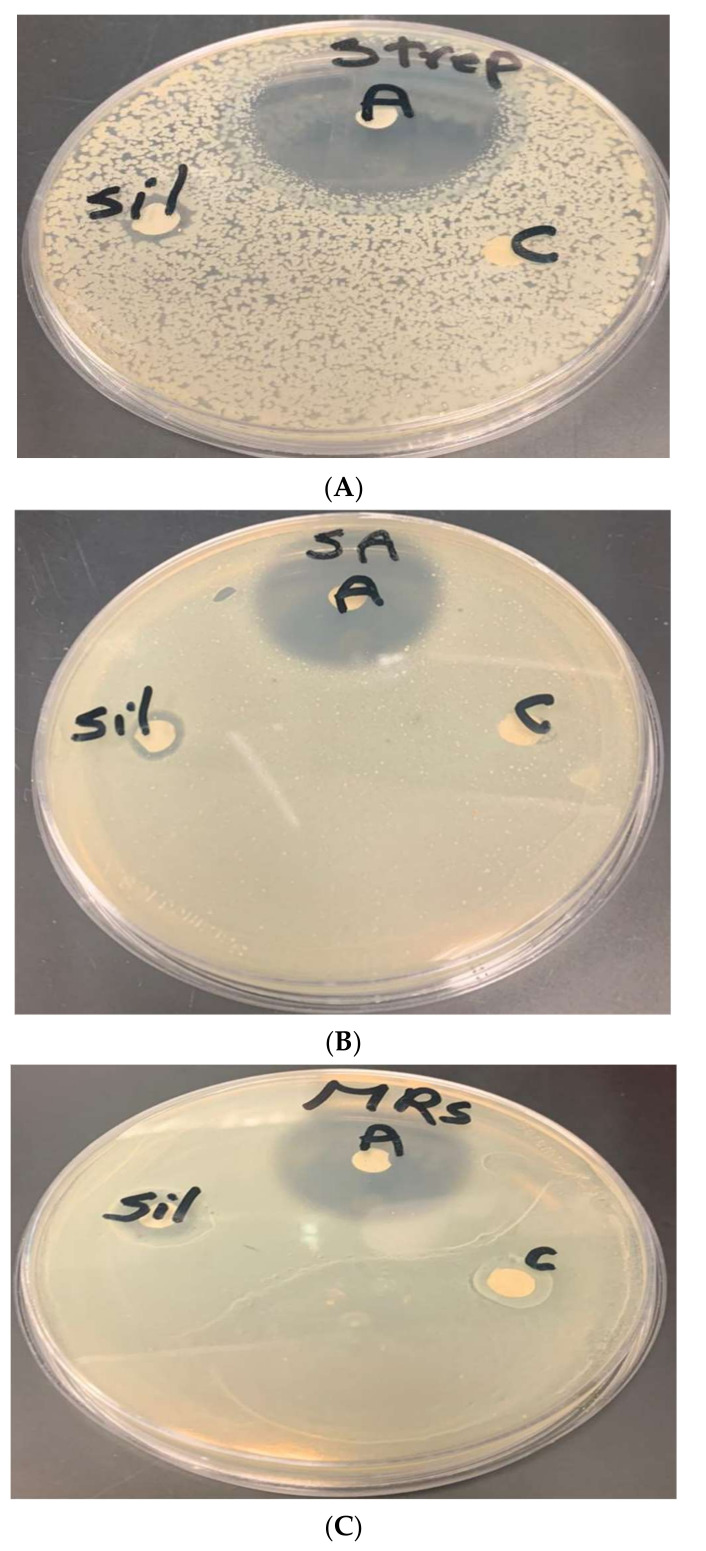
Inhibition zone of silver nanoparticles of *Chlorella vulgaris* extract against (**A**) *Streptococcus* sp., (**B**) *Staphylococcus aureus*, and (**C**) methicillin-resistant *Staphylococcus aureus*.

**Figure 9:**
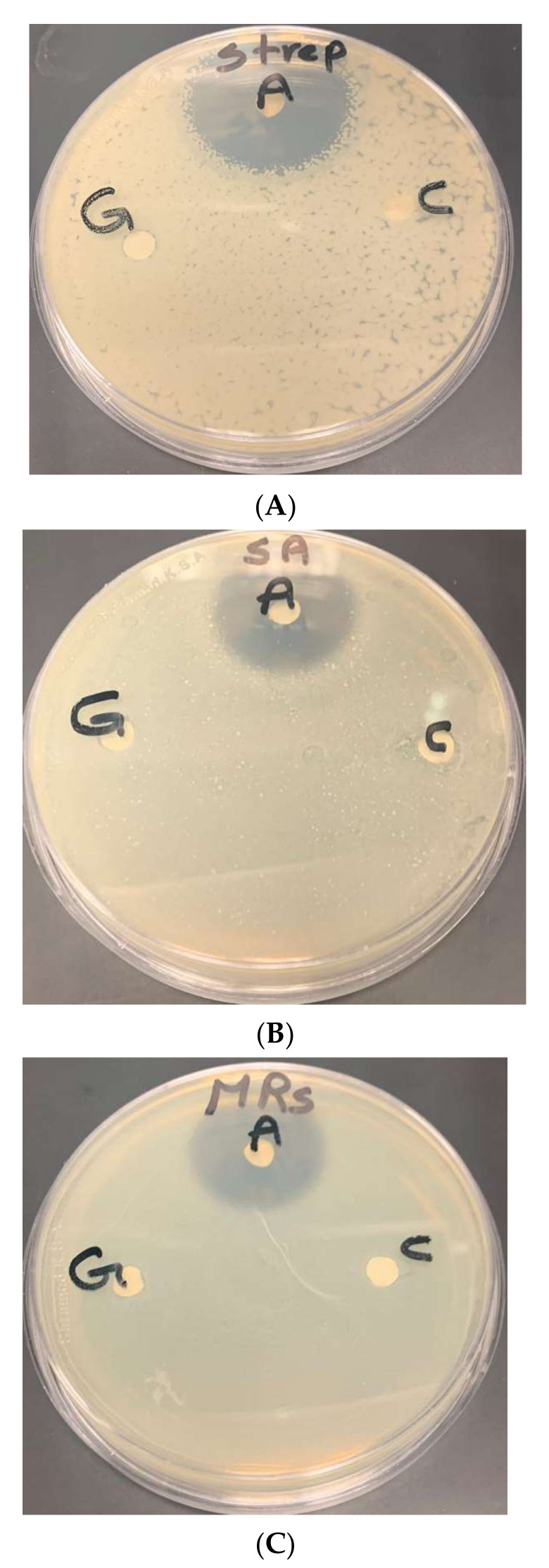
Inhibition zone of gold nanoparticles of *Chlorella vulgaris* extract against (**A**) *Streptococcus* sp., (**B**) *Staphylococcus aureus*, and (**C**) methicillin-resistant *Staphylococcus aureus*.

**Figure 10:**
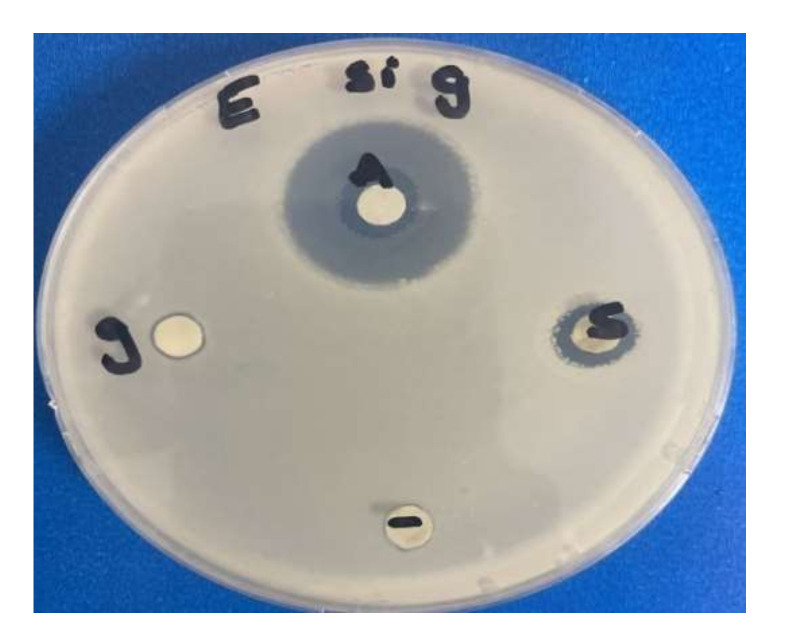
Inhibition zone of silver and gold nanoparticles of *Chlorella vulgaris* against *E. coli*.

The antimicrobial bioassay results (Table 1) showed that the greatest inhibition zone was obtained for *methicillin-resistant staphylococcus aureus* whereas the smallest was for *Streptococcus sp*. (Figure 8). However, despite using low concentration silver of nanoparticles, they showed antimicrobial effect against the multidrug resistant strain of methicillin-resistant *Staphylococcus aureus*. The inhibition zone was 1.7 cm, whereas there were no obvious results on gold nanoparticles compared to silver nanoparticles in the same species (Figure 9). Gram negative *E. coli* growth was also inhibited (Figure 10).

The discs labelled “A” denotes antibiotics; “Sil” denotes disc containing silver nanoparticles; and “c” denotes control disc (containing no silver nanoparticles).

The discs labelled “A” denotes antibiotics; “G“ denotes disc containing gold nanoparticles; and “c” denotes control disc (containing no gold nanoparticles).

The discs labelled “A” denotes antibiotics; “g” denotes disc containing gold nanoparticles; and “s” denotes silver nanoparticles (whereas “−“denotes control disc (containing no nanoparticles)).

### 3.6. Antimicrobial Bioassay Using MIC/MBC µg/mL

The silver and gold nanoparticle showed antibacterial activities with both bacteriostatic and bactericidal capabilities (Table 2). However, some variances were detected amongst the action of the silver and gold nanoparticle compounds. The silver nanoparticle was the most active, while gold nanoparticle showed the lowest activity.

## 4. Discussion

Both nanosilver and nanogold particles showed the characteristic surface plasmon bands that conform with those reported in [16] for nanosilver and in [17] for nanogold. Moreover, the FTIR confirmed the formation of nanogold where the characteristic bands were detected that are attributed to carbonyl stretch and free-amide stretch vibrations in the amide linkages of the proteins. The carbonyl group and peptides of proteins have the ability to bind metal, such that the proteins can act as capping and stabilizing agent of gold nanoparticles to prevent their agglomeration. With regard to the bands of silver nanoparticles, they showed the characteristic peak as that reported by Al dayel et al. [18]. In agreement with this, *Chlorella vulgaris* has been reported to produce nano-silver [19]. However, the scanning electron microscopy showed the tendency of the silver nanoparticles to aggregate indicating the absence of enough stabilizers in the outer external solution. Unlike FTIR which was performed on dried cellular nanoparticles that showed the binding and association with several biomolecules and functional groups. This only indicates that incase of biogenic synthesis of silver nanoparticles, the intracellular nanoparticles are most likely to be stabilized and capped by several cellular bioactive molecules (unlike the extracellular nanosilver, which most likely tends to aggregate due to lack of external stabilizers).

It is worth noting that, unlike cyanobacteria which can secrete stabilizing and capping materials into external solution, the green alga *Chlorella vulgaris* is not able to do so.

Unlike silver nanoparticles, the nanogold particles were more uniform and homogenous and far less prone to aggregation as SEM revealed.

Inorganic nanoparticles of silver and gold are being used in many pharmaceutical applications due to their functionality and biocompatibility [20]. For example, they can be used for their antimicrobial action [21]). The reduction of gold ions is suggested to follow similar path to that to of silver ions, which can proceed through the reductase enzymes [22] and electron carriers such as quinones. Gold nanoparticles are also known for their potent antibacterial activity against acne or scurf and have commercial applications [23]. Zhang et al. (2015) have also reported gold nanoparticle-mediated growth inhibition of different Gram-positive and Gram-negative bacteria and fungi [23].

The pathogenic bacteria used in the antimicrobial bioassay are all of different characteristics and it is quite difficult to find one antimicrobial agent that has broad-spectrum activity against all of them. Indeed, the antimicrobial capability of both nanogold and nano silver particles biosynthesized by *Chlorella* vulgaris was experimentally proven. Nevertheless, the nanosilver particles were more potent antimicrobials than the nanogold particles. Moreover, the antibacterial action of biogenic nano-silver particles was effective against multidrug resistant *Staphylococcus aureus* (MRSA). Skulberg (2000) [24] illustrated that the need to exploit microalgae from rather underexplored areas for their applicable potential as they possess unique metabolites and bioactive compounds of wide array of utilizes and effect that can be effective against multidrug-resistant strains. With regard to their effect on Gram-negative *Escherichia coli*, both showed antimicrobial action but in case of gold nanoparticles it showed weak antibacterial impact unlike nanosilver particles which were more potent antibacterial agents. The cells of Gram-negative bacteria are hard to penetrate since there is a huge outer lipid membrane that protects it. Nonetheless, those cells were susceptible to silver nanoparticles. Overall, silver nanoparticles showed a broad-spectrum antimicrobial bioactivity against different pathogenic bacteria (both Gram-positive and Gram-negative) and even against multidrug-resistant bacterium. This only attests for the great antimicrobial potential of silver nanoparticles.

### The Antimicrobial Bioassay Using Dilution Test

The mechanism of action of silver and gold nanoparticles exhibited both bacteriostatic and bactericidal action through the inhibition of bacterial enzymes by binding to DNA, as well as causing damage to both the bacterial cell wall and cytoplasmic membrane [25]. However, when comparing the antimicrobial effect of silver nanoparticles to that of gold nanoparticles, the superiority of silver nanoparticles is most evident with its wide-spectrum antimicrobial action against both Gram-positive and Gram-negative bacteria. In addition, the outer solution of *Chlorella vulgaris* may have numerous bioactive compounds such as flavonoids, tannin, phenolic compounds, terpenes, glycosides, and saponins (which has been reported, for example, by [26]. Those compounds may act as stabilizers/capping agents for those nanoparticles present in the outer solution of *Chlorella* sp. cells.

The biosynthetic mechanism of these nanoparticles is debatable and is thought to proceed both biotically via enzymes and abiotically by cellular reducing entities. Nonetheless, we detected the nearly complete loss of vitality, as indicated from the disintegration of total RNA, after four hours of incubation with silver nitrate and the complete loss of vitality for periods longer than this. Nonetheless, the accumulation of silver nanoparticles continued which is most likely through the abiotic pathway.

Overall, *Chlorella vulgaris* is a promising bio-source for antimicrobials most important of which are biogenic nanoparticles as silver nanoparticles. Nonetheless, the accumulation of these nanoparticles seems to be lethal to the algal itself but the accumulation of nanoparticles continues after its mortality possibly via functional groups within the non-living biomass. These results undoubtedly verify the strong antimicrobial potency of biogenic silver nanoparticles. Given the other merits of their green, cost-free, and easy synthesis, biogenic nanosilver represents the most plausible candidate for antimicrobial pharmaceuticals.

Therefore, we recommend the mass exploitation of the algal biomass for the purpose of nanoparticles pharmaceutical production.

## Figures and Tables

**Table 1:** Antibacterial activity of silver and gold nanoparticles of *Chlorella vulgaris*.

Bacterial Species	Inhibition Zone for Nanogold	Inhibition Zone for Nanosilver
*Staphylococcus aureus*	0.4 ± 0.2	1.2 ± 0.1
*Streptococcus* sp.	0.5 ± 0.1	1.0 ± 0.2
Methicillin-resistant *Staphylococcus aureus*	0.3 ± 0.1	1.7 ± 0.1
*Escherichia coli*	0.9 ± 0.1	1.9 ± 0.1

**Table 2:** Antimicrobial activity of silver and gold nanoparticle (MIC/MBC µg/mL). MIC stands for minimum inhibitory concentration whereas MBC stands for minimum bactericidal concentration.

Bacteria	Nanosilver	Nanogold	Chloamphenicol 5%
*E. coli*	62.5/125	125/250	62.5/125
*S. aureus*	125/250	>250	62.5/125
*Streptococcus* sp.	250/250	>1000	>250
Methicillin Resistant *Staphylococcus aureus*	125/250	250/250	62.5/125

## Data Availability

All data generated or analyzed during this study are included in this published article.

## Abbreviations

AgNPs	Silver nanoparticles
KSA	Kingdom of Saudi Arabia
MRSA	Methicillin-resistant *Staphylococcus aureus*
DNA	Deoxyribonucleic acid
